# Effects of NS *lactobacillus* strains on lipid metabolism of rats fed a high-cholesterol diet

**DOI:** 10.1186/1476-511X-12-67

**Published:** 2013-05-09

**Authors:** Xu Hu, Tao Wang, Wei Li, Feng Jin, Li Wang

**Affiliations:** 1 Key Laboratory of Mental Health, Institute of Psychology, Chinese Academy of Sciences, Beijing, China; 2 University of Chinese Academy of Sciences, Beijing, China; 3 School of Medicine, Hangzhou Normal University, Zhejiang, China

**Keywords:** NS *lactobacillus*, Cholesterol, Apolipoprotein, Intestinal microbiota, mRNA Expression

## Abstract

**Background:**

Elevated serum cholesterol level is generally considered to be a risk factor for the development of cardiovascular diseases which seriously threaten human health. The cholesterol-lowering effects of lactic acid bacteria have recently become an area of great interest and controversy for many researchers. In this study, we investigated the effects of two NS *lactobacillus* strains, *Lactobacillus plantarum* NS5 and *Lactobacillus delbrueckii* subsp. *bulgaricus* NS12, on lipid metabolism of rats fed a high cholesterol diet.

**Methods:**

Thirty-two SD rats were assigned to four groups and fed either a normal or a high-cholesterol diet. The NS *lactobacillus* treated groups received the high-cholesterol diet supplemented with *Lactobacillus plantarum* NS5 or *Lactobacillus delbrueckii* subsp. *bulgaricus* NS12 in drinking water. The rats were sacrificed after a 6-week feeding period. Body weights, visceral organ and fat weights, serum and liver cholesterol and lipid levels, intestinal microbiota and liver mRNA expression levels related to cholesterol metabolism were analyzed. Liver lipid deposition and adipocyte size were evaluated histologically.

**Results:**

Compared with rats fed a high cholesterol diet, serum total cholesterol, low-density lipoprotein cholesterol, apolipoprotein B and free fatty acids levels were decreased and apolipoprotein A-I level was increased in NS5 or NS12 strain treated rats, and with no significant change in high-density lipoprotein cholesterol level. Liver cholesterol and triglyceride levels were also significantly decreased in NS *lactobacillus* strains treated groups. Meanwhile, the NS *lactobacillus* strains obviously alleviated hepatic injuries, decreased liver lipid deposition and reduced adipocyte size of high cholesterol diet fed rats. NS *lactobacillus* strains restored the changes in intestinal microbiota compositions, such as the increase in *Bacteroides* and the decrease in *Clostridium*. NS *lactobacillus* strains also regulated the mRNA expression levels of liver enzymes related to cholesterol metabolism, including the down regulation of acyl-CoA:cholesterol acyltransferase (ACAT) and the upregulation of cholesterol 7α-hydroxylase (CYP7A1).

**Conclusion:**

This study suggested that the two NS *lactobacillus* strains may affect lipid metabolism and have cholesterol-lowering effects in rats fed a high cholesterol diet.

## Introduction

The incidence of hypercholesterolemia is increasing rapidly with the improvements of people’s living standards and alterations of lifestyles. Hypercholesterolemia is characterized by very high levels of cholesterol in the blood. Elevated serum cholesterol level is generally considered to be the most important risk factor for the development of cardiovascular diseases (CVDs), such as hypertension, coronary heart disease, diabetes. The WHO has predicted that by 2030, approximately 23.6 million people will die from CVDs and these are projected to remain the single leading causes of death [1]. It has been reported that even a 1% decrease in serum cholesterol levels could lower the risk of coronary heart disease up to 3% [2]. Therefore, lowering serum cholesterol levels is one of the effective means for the prevention of CVDs. There are many commonly used cholesterol lowering drugs, such as statins are widely used in clinical practice to lower the serum cholesterol levels [3]. However, because of the high prices and side effects of the commonly used drugs, it is more and more attractive to develop more effective and safer alternative therapies to lower serum cholesterol.

Studies showed that the number and species of intestinal microbiota played an important role in maintaining normal physiological states of biological organisms and intestinal microbiota perturbations may potentially lead to disease states [4]. Stepankova *et al*. found that the absence of microbiota (germ-free conditions) accelerated the atherosclerosis in ApoE-deficient mice fed standard low cholesterol diet [5]. Studies also found that the gut microbiota modulated lipid metabolism in mice. The level of cholesterol in conventionally raised mice was lower than that of germ-free mice [6]. These facts indicated the existence of the relationship between intestinal microbiota and hypercholesterolemia. Therefore, effective interventions in intestinal microbiota may be able to achieve the purpose of reducing serum cholesterol level. Lactic acid bacteria (LAB) are widely distributed in human intestinal and considered to play beneficial effects on human health by modulating intestinal microbiota balance [7]. LAB supplementation may be able to reduce serum cholesterol level. Early in 1974, Mann and Spoerry firstly discovered the cholesterol-lowering effects of fermented milk ingested by the Massai tribes people [8]. Since then, more and more evidences have suggested the effects of LAB on serum cholesterol levels in animal models [9-14] and humans [15-19]. However, conflicting results have been obtained from different experiments [20,21]. Therefore, the serum cholesterol-lowering effects of probiotics are still in debating and the precise mechanisms have not been clearly elucidated. Many possible mechanisms have been proposed to explain the hypocholesterolemic effects of LAB, such as assimilation of cholesterol by LAB [22], binding cholesterol to the bacterial cellular surface [23], incorporating cholesterol into the bacterial cellular membranes [24], converting into coprostanol by cholesterol reductase produced by LAB [25-27]. Based on the biological feature of LAB in intestine, we proposed other new possibility that LAB display the cholesterol-lowering effects by means of modulating the compositions of intestinal microbiota.

The health benefits of LAB are strain specific and different strains display different beneficial effects [28]. Based on these, a series of *lactobacillus* strains were isolated from the natural fermented dairy products collected from grassland in Inner Mongolia of China by our laboratory and were named NS *lactobacillus* strains. Firstly, through *in vitro* experiment, we screened the strains and found two of them with the highest cholesterol-lowering abilities, *Lactobacillus plantarum* NS5 and *Lactobacillus delbrueckii* subsp. *bulgaricus* NS12. In this study, we examined the effects of the two NS *lactobacillus* strains on serum and liver lipid levels in high-cholesterol diet fed male SD rats, and explored the possible mechanisms from the aspects of intestinal microbiota compositions and mRNA expression levels of liver enzymes related to cholesterol metabolism.

## Materials and methods

### NS *lactobacillus* strains used in this study

The NS *lactobacillus* strains used in this study, *Lactobacillus plantarum* NS5 [GenBank accession no. JQ013297] and *Lactobacillus delbrueckii* subsp. *bulgaricus* NS12 [GenBank accession no. JX839763], were two of the strains isolated from the natural fermented dairy products collected from grassland in Inner Mongolia of China by our laboratory. They showed better abilities to lower cholesterol in *in vitro* trials. They were individually inoculated into MRS media and incubated at 37°C for 24 hours. Then the *lactobacillus* strains were collected by centrifugation at 3000 rpm for 10 minutes and washed twice with normal saline (0.9% NaCl). The strains were resuspended in sterile water and used as drinking water at a concentration of 10^8^ CFU/ml (The drinking water was changed every day, 8–10 ml of water was consumed per 100 g rat body weight per day).

### Animal groups and diets

Thirty-two male Sprague–Dawley rats, conventional clean animal grade and aged 3 weeks, were purchased from Vital River Laboratory Animal Ltd. (Beijing, China). They were fed a normal diet (Keao Xieli Feed Co. Ltd., Beijing, China) for 1 week. Food and water were supplied ad libitum. Rats were individually housed in cages and maintained at a constant temperature (23 ± 2°C) and humidity (55 ± 5%) and exposed to a 12-h light/dark cycle. The animals were cared for in accordance with *the Guiding Principles in the Care and Use of Animals*. All experiments were approved by the Institutional Animal Care and Use Committee of the Institute of Psychology of the Chinese Academy of Sciences.

After 1-week adaptive period on a normal diet containing 32% (weight/weight) protein, 5% fat, 2% fiber, 1.8% calcium, 1.2% phosphorus, and a nitrogen-free extract as the remainder, the 32 rats were randomly assigned to four groups of 8 rats each. The four groups were assigned diets as follows: (1) Control group, normal diet; (2) HC group, high-cholesterol diet; (3) HC + NS5 group, high-cholesterol diet with *Lactobacillus plantarum* NS5 added in drinking water; (4) HC + NS12 group, high-cholesterol diet with *Lactobacillus delbrueckii* subsp. *bulgaricus* NS12 added in drinking water. The high-cholesterol diet contained 2% (weight/weight) cholesterol, 10% lard, 0.3% sodium cholate and 87.7% normal diet. The rats were fed for 6 weeks and body weight was recorded. After the feeding period, the rats were euthanized and the weight of visceral organs (heart, liver, spleen, lung and kidney) and mesenteric, perirenal and epididymal white adipose tissues (WAT) were measured.

### Assay for blood serum indexes

Before the experiment period (week 0) and at the end of week 2 and week 4 of the experiment period, the rats were deprived of food for 12 hours, and then 100 μl blood samples were collected with capillary tubes from the tail vein and were used to analyze the serum cholesterol content. By the end of the experiment period (week 6), about 5 ml blood samples from each rat were collected into tubes using cardiac puncture following food deprivation for 12 hours. The serum was separated from the blood by centrifugation at 3500 rpm for 10 minutes. The total cholesterol (TC), high density lipoprotein cholesterol (HDL-C), low density lipoprotein cholesterol (LDL-C) were measured with automatic chemical analyzer. Serum free fatty acids were estimated using an ultrasensitive assay kit for free fatty acids (Applygen Technologies Inc., Beijing). Apolipoprotein A-I (ApoA-I) and apolipoprotein B (ApoB) were analyzed with rat ApoA-I and ApoB ELISA kit (Bio-function Technology Ltd., Beijing) according to the manufacturer’s recommended protocol.

### Assay for liver TC and TG

After the rats had been killed, the livers were removed and rinsed with physiological saline solution, blotted dry with filter paper and weighed. Liver total cholesterol (TC) and triglyceride (TG) contents were determined using tissue total cholesterol and triglyceride assay kit (Applygen Technologies Inc., Beijing) according to the manufacturer’s instructions.

### Histopathology of liver and adipocytes

After euthanasia, rat livers and adipocytes were removed and frozen rapidly to about −20 to −30°C. The specimens were then embedded in optimal cutting temperature (OCT) compound (Sakura Finetek USA Inc., USA) and subsequently they were cut frozen with Leica CM1900 Cryostat. The sections were picked up on a glass slide and stained. The liver specimens were stained with hematoxylin-eosin (HE) or oil red O. The adipocytes specimens were also stained with HE and adipocyte numbers were counted in 3 different spots per rat.

### Intestinal microbiota analysis

Fecal samples were collected at sacrifice and frozen at −20°C. Bacterial DNA was extracted using a TIANamp stool DNA kit (Tiangen Biotech Co. Ltd., Beijing, China) following the manufacturer’s instructions. DNA was amplified by qPCR using SYBR® Premix Ex Taq™ (Takara, Japan) and primer sets (Table 1) designed for 16S rRNA of bacterial species including *Eubacteria* (all bacteria; used as housekeeping gene), *Firmicutes*, *Bacteroides* and *Clostridium* to analyze intestinal microbiota between different groups. Results were presented as percentage expression of each species relative to total bacteria (*Eubacteria*).

**Table 1:** Primer sequences used in this study

**Target**	**Forward (5′-3′)**	**Reverse (5′-3′)**
Intestinal microbiota		
*Eubacteria*	ACTCCTACGGGAGGCAGCAGT	ATTACCGCGGCTGCTGGC
*Firmicutes*	GGAGYATGTGGTTTAATTCGAAGCA	AGCTGACGACAACCATGCAC
*Bacteroides*	GAGAGGAAGGTCCCCCAC	CGCTACTTGGCTGGTTCAG
*Clostridium*	ACTCCTACGGGAGGCAGC	GCTTCTTAGTCAGGTACCGTCAT
mRNA		
GAPDH	GCAAGTTCAACGGCACAG	CGCCAGTAGACTCCACGAC
HMG-CoA R	TGTGGGAACGGTGACACTTA	CTTCAAATTTTGGGCACTCA
LDLR	AGCCGATGCATTCCTGACTC	AGTTCATCCGAGCCATTTTCAC
CYP7A1	ACGTGGTTGGAAGAAGCG	GAATGTGGGCAGCGAGAA
ACAT	GCTGAAGTGAACTACCCCTT	GAGCCATGCCTCTAGTACCT
LCAT	CCCAAGGCTGAACTCAGTAACCA	CGGTAGCACAGCCAGTTTACCA

### mRNA expression levels

The mRNA expression levels of liver enzymes associated with cholesterol metabolism were analyzed. Total RNA was isolated from rat liver by TRIZOL reagent (Tiangen Biotech Co. Ltd., Beijing, China) according to the manufacturer’s protocol. The RNA concentration was estimated by measurement with a UV spectrophotometer at 260 and 280 nm and transcribed into cDNA (Tiangen Biotech Co. Ltd., Beijing, China). The mRNA expression of genes was measured by RT-PCR using SYBR® Premix Ex Taq™ (Takara, Japan), with values presented as 2^-ΔΔCT^. We used the housekeeping gene for glyceraldehyde-3-phosphate dehydrogenase (GAPDH; EC1.2.1.12) for normalization. The genes and primer sequences used for RT-PCR are described in Table 1.

### Statistical analysis

All experimental data were presented as the mean ± standard deviations (SD). Data were analyzed using a one-way analysis of variance using SPSS 17.0 statistical software (SPSS Inc., Chicago, IL, USA); Values of p < 0.05 were considered statistically significant.

## Results

### The growth of rats and the weight of visceral organs

At the beginning and end of the experimental period, the body weight of rats in all four groups was recorded. All the rats used in this study appeared healthy throughout the whole feeding period. The initial body weight of rats showed no significant difference between groups. After 6 weeks of experimental period, the rats in HC group which fed a high-cholesterol diet exhibited an increasing trend in body weight compared with control group which fed a normal diet, but not yet reached to statistical significance (441.9 ± 16.3 *vs*. 415.7 ± 30.1 g, p = 0.070). Adding 10^8^ CFU/ml of *Lactobacillus plantarum* NS5 (HC + NS5 group) or *Lactobacillus delbrueckii* subsp. *bulgaricus* NS12 (HC + NS12 group) in drinking water, they showed a slight decrease in final body weight compared with HC group, but also without significant difference (Table 2).

**Table 2:** Body weight changes of rats between groups after 6 weeks

**Group**	**Rats (n)**	**Initial weight (g)**	**Final weight (g)**
Control	8	89.4 ± 10.3	415.7 ± 30.1
HC	8	90.3 ± 7.9	441.9 ± 16.3
HC + NS5	8	89.4 ± 7.5	426.8 ± 24.6
HC + NS12	8	90.7 ± 8.3	428.0 ± 22.6

Control: normal diet; *HC*, high cholesterol diet; *HC* + *NS5*, high cholesterol diet + *Lactobacillus plantarum NS5*; *HC* + *NS12*, high cholesterol diet + *Lactobacillus delbrueckii* subsp. *bulgaricus* NS12.

The data are shown as the mean ± standard deviation.

After the rats were killed, the visceral organs (heart, liver, spleen, lung and kidney) and mesenteric, perirenal and epididymal white adipose tissues (WAT) were separated and weighed (Table 3). The rats had the lowest liver weight in control group and had the highest liver weight in HC group. *Lactobacillus plantarum* NS5 significantly lowered liver weight as compared with the HC group (p < 0.05). *Lactobacillus delbrueckii* subsp. *bulgaricus* NS12 also lowered the liver weight, but without statistical significance (p = 0.064). There were no significant differences in the weight of hearts, spleens, lungs and kidneys among the four groups. Similarly, control group had the lowest WAT weight and HC group had the highest WAT weight, but not yet reached to statistical significance.

**Table 3:** Effect of NS5 and NS12 on visceral organs and WAT weight in rats

**Weight (g)**	**Control (n = 8)**	**HC (n = 8)**	**HC + NS5 (n = 8)**	**HC + NS12 (n = 8)**
Heart	1.5 ± 0.2	1.5 ± 0.1	1.5 ± 0.2	1.4 ± 0.2
Liver	14.4 ± 1.4^a^	22.2 ± 3.1^b^	18.7 ± 2.1^c^	19.3 ± 3.7^bc^
Spleen	0.9 ± 0.2	0.9 ± 0.1	0.7 ± 0.3	0.8 ± 0.2
Lung	2.3 ± 0.3	2.3 ± 0.3	2.3 ± 0.2	2.2 ± 0.4
Kidney	3.4 ± 0.7	3.2 ± 0.4	3.0 ± 0.3	3.3 ± 0.7
Epididymal fat	4.7 ± 1.3	6.2 ± 1.2	5.2 ± 1.2	4.9 ± 1.5
Perirenal fat	6.6 ± 1.6	8.2 ± 2.1	7.2 ± 2.4	6.5 ± 1.7
Mesenteric fat	5.5 ± 1.1	6.5 ± 1.0	5.8 ± 0.9	5.5 ± 1.8

Control: normal diet; *HC*, high cholesterol diet; *HC* + *NS5*, high cholesterol diet + *Lactobacillus plantarum NS5*; *HC* + *NS12*, high cholesterol diet + *Lactobacillus delbrueckii* subsp. *bulgaricus* NS12.

The data are shown as the mean ± standard deviation.

^a,b,c^ Mean values within a row with different superscript letters differ significantly (p < 0.05).

### Effect of NS5 and NS12 strains on serum lipids

**Figure 1:**
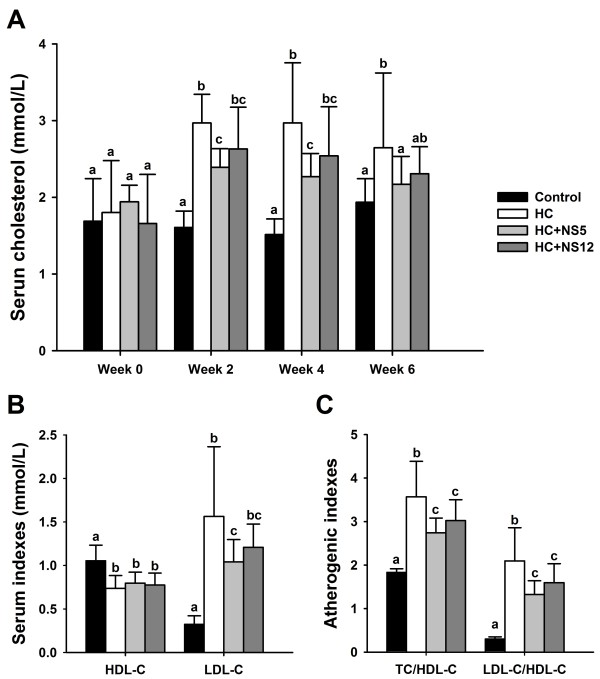
**Effects of the two NS *lactobacillus *strains on serum cholesterol in rats fed a high cholesterol diet.** (**A**) Serum total cholesterol content; (**B**) Serum HDL-C and LDL-C; (**C**) Atherogenic indexes of the rats, TC/HDL-C and LDL-C/HDL-C ratios. The data are shown as the mean ± standard deviation. Control: normal diet; HC: high cholesterol diet; HC + NS5: high cholesterol diet + *Lactobacillus plantarum* NS5; HC + NS12: high-cholesterol diet + *Lactobacillus delbrueckii* subsp. *bulgaricus* NS12. ^a,b,c^ Mean values in each panel with different superscript letters differ significantly (p < 0.05).

Blood serum total cholesterol (TC) levels in the four groups were shown in Figure 1A. Before the experiment, serum TC showed no significant difference between groups. High-cholesterol diets could significantly increase serum TC levels compared with control group. *Lactobacillus plantarum* NS5 and *Lactobacillus delbrueckii* subsp. *bulgaricus* NS12 showed varying degrees of cholesterol-lowering abilities *in vivo*. Compared with the HC group, NS5 strain significantly lowered the serum TC levels. At the end of 2 weeks and 4 weeks, the serum TC levels in HC + NS5 group were still different from control group with statistical significance, but at the end of 6 weeks, there was even no significant difference compared with control group. NS12 strain also reduced the serum TC levels to some extent compared with HC group, but without statistical significance. However, there was also no significant difference between HC + NS5 and HC + NS12 groups.

The control group displayed the highest HDL-C level, and high-cholesterol diets reduced the serum HDL-C concentration. But the NS5 and NS12 strains did not show obvious influences on HDL-C levels compared with HC group. However, the elevated serum LDL-C levels induced by feeding high-cholesterol diets were reduced in the NS5 and NS12 strains treated groups. HC + NS5 group showed a statistical significant difference and the difference was not significant in HC + NS12 group (Figure 1B). Atherogenic indexes (TC/HDL-C and LDL-C/HDL-C ratios) were calculated (Figure 1C). The HC group demonstrated the highest and the control group demonstrated the lowest atherogenic indexes in all the four groups (TC/HDL-C: 3.57 ± 0.82 vs. 1.84 ± 0.08, p < 0.05 and LDL-C/HDL-C: 2.10 ± 0.76 vs. 0.30 ± 0.05, p < 0.05). The NS5 and NS12 strains significantly lowered the TC/HDL-C and LDL-C/HDL-C ratios comparing with the HC group. Although NS5 strains seemed more effective than NS12 strains, there were no significant differences in lowering the atherogenic indexes between the two groups.

**Figure 2:**
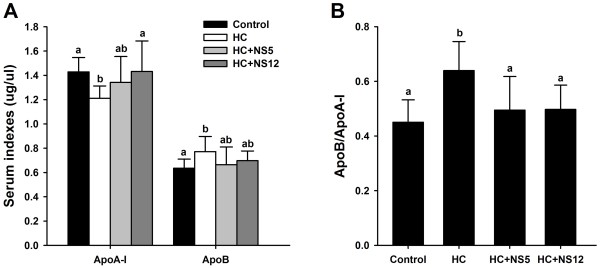
**Effects of the two NS *lactobacillus *strains on serum apolipoprotein in rats fed a high cholesterol diet.** (**A**) Serum ApoA-I and ApoB; (**B**) Serum ApoB/ApoA-I ratio. The data are shown as the mean ± standard deviation. Control: normal diet; HC: high cholesterol diet; HC + NS5: high cholesterol diet + *Lactobacillus plantarum* NS5; HC + NS12: high-cholesterol diet + *Lactobacillus delbrueckii* subsp. *bulgaricus* NS12. ^a,b^ Mean values in each panel with different superscript letters differ significantly (p < 0.05).

Apolipoprotein A-I(ApoA-I) and apolipoprotein B (ApoB) in serum were also analyzed. High-cholesterol diets significantly lowered the serum ApoA-I level and increased the ApoB level (Figure 2A). The NS12 strain significantly increased the serum ApoA-I level compared with HC group (p < 0.05) and with no difference from control group (p > 0.05). The rise of ApoA-I in HC + NS5 group was not statistical significant compared with HC group (p > 0.05). However, they were also not significantly different from control group and HC + NS12 group. NS5 and NS12 strains displayed a trend to decrease the ApoB level comparing with HC group, but there were no statistical significance. Nevertheless, there were also no differences compared with control group. ApoB1/ApoA-I ratio was also calculated and the rats in HC group had the highest ApoB/ApoA-I ratio in all four groups. NS5 and NS12 strains could decrease the ratios compared with HC group (Figure 2B).

**Figure 3:**
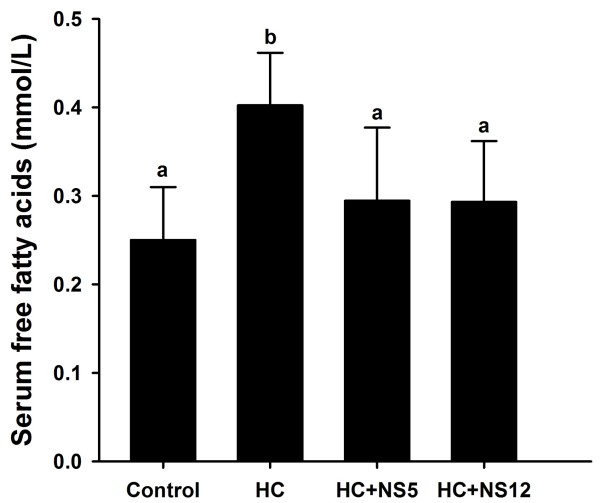
**Effects of the two NS *lactobacillus *strains on serum free fatty acids in rats fed a high cholesterol diet.** The data are shown as the mean ± standard deviation. Control: normal diet; HC: high cholesterol diet; HC + NS5: high cholesterol diet + *Lactobacillus plantarum* NS5; HC + NS12: high-cholesterol diet + *Lactobacillus delbrueckii* subsp. *bulgaricus* NS12. ^a,b^ Mean values in each panel with different superscript letters differ significantly (p < 0.05).

Serum free fatty acids (FFA) were also significantly increased after feeding high-cholesterol diets. Adding NS5 and NS12 strains to drinking water could lower the serum FFA levels to the extent that have no difference with control group (Figure 3).

### NS5 and NS12 strains reduced liver TC and TG levels in rats fed a high-cholesterol diet

**Figure 4:**
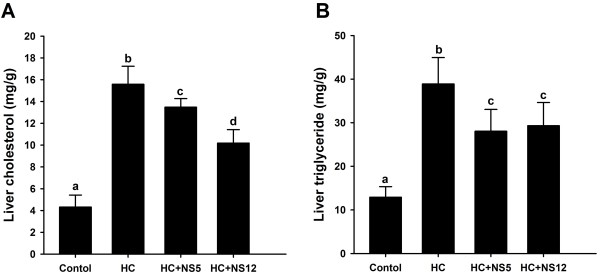
**Effects of the two NS *lactobacillus *strains on liver cholesterol and triglyceride in rats fed a high cholesterol diet.** (**A**) Liver total cholesterol; (**B**) Liver triglyceride. The data are shown as the mean ± standard deviation. Control: normal diet; HC: high cholesterol diet; HC + NS5: high cholesterol diet + *Lactobacillus plantarum* NS5; HC + NS12: high-cholesterol diet + *Lactobacillus delbrueckii* subsp. *bulgaricus* NS12. ^a,b,c,d^ Mean values in each panel with different superscript letters differ significantly (p < 0.05).

Liver TC levels differed significantly among the four groups (Figure 4A). The liver TC levels of rats fed a high-cholesterol diet had greatly increased compared with control group. NS5 and NS12 strains could significantly reduce the liver TC levels comparing with HC group; and NS12 was more effective than NS5.

The control group displayed the lowest liver TG level and HC group displayed the highest liver TG level. Both the HC + NS5 and HC + NS12 groups had lower levels of liver TG than HC group (p < 0.05) and there were no obvious differences between the two NS *lactobacillus* groups (Figure 4B).

### Histopathology of liver and adipocytes

**Figure 5:**
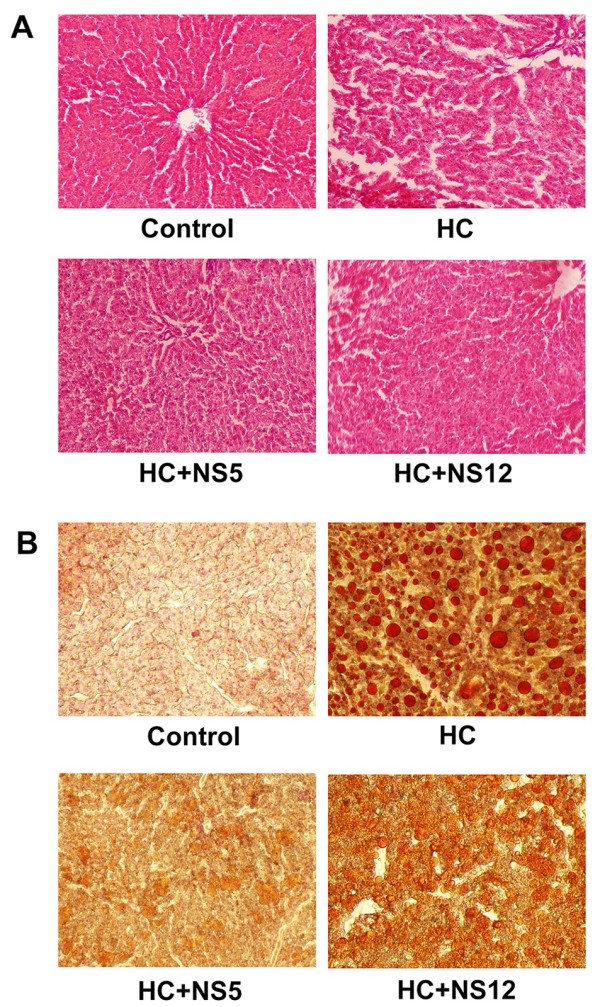
**Effects of the two NS *lactobacillus *strains on histology of liver in rats fed a high cholesterol diet.** (**A**) Representative photomicrographs of hematoxylin and eosin staining (100×); (**B**) Representative photomicrographs of oil red O staining (200×). Control: normal diet; HC: high cholesterol diet; HC + NS5: high cholesterol diet + *Lactobacillus plantarum* NS5; HC + NS12: high-cholesterol diet + *Lactobacillus delbrueckii* subsp. *bulgaricus* NS12.

Figure 5 illustrated the effects of *Lactobacillus plantarum* NS5 and *Lactobacillus delbrueckii* subsp. *bulgaricus* NS12 on hepatic steatosis. Hematoxylin-eosin (HE) and oil red O staining showed the differences in liver tissue structures and lipids accumulation of the four groups. The rat livers of control group had a well-organized structure. Hepatic sinusoids were clearly visible and hepatic cords were neatly arranged and distributed radially around the central veins. The structures of livers displayed large degrees of damages in HC group, and hepatocytes showed signs of necrosis. Hepatocyte steatosis was obviously alleviated by adding NS5 and NS12 strains to drinking water compared with HC group (Figure 5A). Oil red O staining suggested that many massive lipid droplets were accumulated in the liver tissues in HC group and the lipid droplets had obviously decreased in HC + NS5 and HC + NS12 groups (Figure 5B).

**Figure 6:**
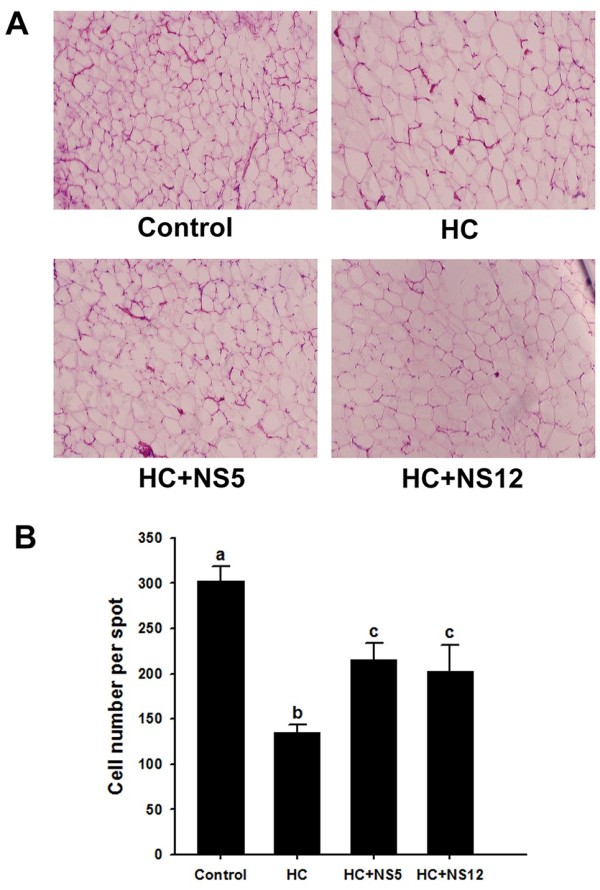
**Effects of the two NS *lactobacillus *strains on adipocyte size in rats fed a high cholesterol diet.** (**A**) Representative photomicrographs of hematoxylin and eosin staining of adipocytes (200×); (**B**) Adipocyte numbers in 3 different spots per rat. The data are shown as the mean ± standard deviation. ^a,b,c^ Mean values in each panel with different superscript letters differ significantly (p < 0.05). Control: normal diet; HC: high cholesterol diet; HC + NS5: high cholesterol diet + *Lactobacillus plantarum* NS5; HC + NS12: high cholesterol diet + *Lactobacillus delbrueckii* subsp. *bulgaricus* NS12.

The rats in HC group demonstrated increased adipocyte size comparing with control groups (Figure 6A). This was proved by judging from the changes in adipocyte number per spot. The more the adipocyte number was, the smaller the adipocyte size was. The adipocyte size was partly restored by NS5 and NS12 strains.

### NS5 and NS12 strains recovered the changes of intestinal microbiota caused by high-cholesterol diets

**Figure 7:**
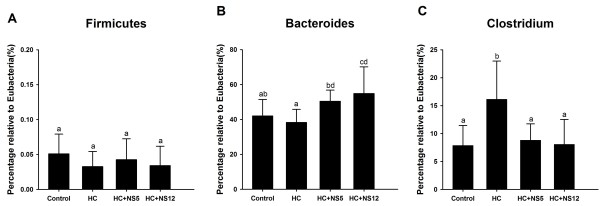
**Effects of the two NS *lactobacillus *strains on intestinal microbiota compositions in rats fed a high cholesterol diet.** (**A**) Percentage of *Firmicutes* relative to Eubacteria; (**B**) Percentage of *Bacteroides* relative to Eubacteria; (**C**) Percentage of *Chostridium* relative to Eubacteria. The data are shown as the mean ± standard deviation. Control: normal diet; HC: high cholesterol diet; HC + NS5: high cholesterol diet + *Lactobacillus plantarum* NS5; HC + NS12: high-cholesterol diet + *Lactobacillus delbrueckii* subsp. *bulgaricus* NS12. ^a,b,c,d^ Mean values in each panel with different superscript letters differ significantly (p < 0.05).

No significant difference was observed in fecal *Firmicutes* of rats in all the four groups (Figure 7A). There was the lowest percentage of *Bacteroides* in feces of rats fed a high-cholesterol diet, but without statistical significance compared with control group. After feeding with NS5 and NS12 strains, the percentage of *Bacteroides* was obviously increased comparing with HC groups (Figure 7B). High-cholesterol diet resulted in an increase in *Clostridium*, which was ameliorated after treated with NS *lactobacillus* strains (Figure 7C). The percentages of *Clostridium* in HC + NS5 and HC + NS12 groups were not different from control group.

### mRNA expression levels of liver enzymes associated with cholesterol metabolism

**Figure 8:**
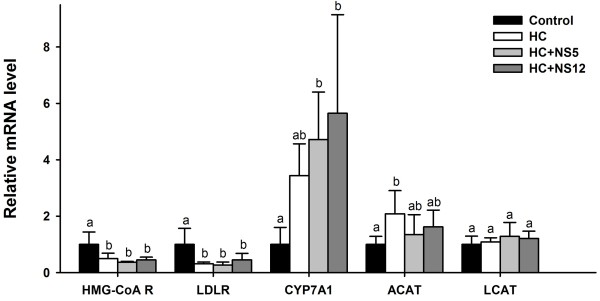
**Effects of the two NS *lactobacillus *strains on mRNA expression levels of liver enzymes associated with cholesterol metabolism in rats fed a high cholesterol diet.** The graph represents the mRNA levels relative to GAPDH. The data are shown as the mean ± standard deviation. Control: normal diet; HC: high cholesterol diet; HC + NS5: high cholesterol diet + *Lactobacillus plantarum* NS5; HC + NS12: high-cholesterol diet + *Lactobacillus delbrueckii* subsp. *bulgaricus* NS12. ^a,b^ Mean values in each panel with different superscript letters differ significantly (p < 0.05).

The mRNA expression levels of hepatic enzymes associated with cholesterol metabolism were illustrated in Figure 8. HMG-CoA reductase (HMG-CoA R) is the rate-limiting enzyme in hepatic cholesterol *de novo* synthesis, catalyzing the conversion of HMG-CoA to mevalonic acid. Its mRNA expression levels were reduced by 50%, 64% and 55% in HC, HC + NS5 and HC + NS12 groups compared to control group, respectively. The HMG-CoA R mRNA level in HC + NS5 group was the lowest. LDLR is a receptor on the hepatocyte surface that mediates the endocytosis of LDL-cholesterol. Its mRNA expression levels were all decreased in the three high-cholesterol diet fed groups and the level of HC + NS12 group was the highest among the three groups. Cholesterol 7α-hydroxylase (CYP7A1) is the enzyme in the synthesis of bile acid from cholesterol. Its mRNA expression levels in HC, HC + NS5 and HC + NS12 groups were 2.44-, 3.72- and 4.65-fold higher than control group respectively; and the HC + NS12 group had the highest level. Acyl-CoA:cholesterol acyltransferase (ACAT) is the enzyme responsible for esterification of cholesterol, and its mRNA expression level in HC group was significantly higher than control group. NS5 and NS12 strains decreased the levels compared with HC group, but there were no statistical significance. However, there were also no differences compared with control group. The lecithin cholesterol acyltransferase (LCAT) mRNA expression in all the four groups were not significantly different.

## Discussion

Cardiovascular diseases (CVDs) are the most important cause of death in the world, including China. Hypercholesterolemia is strongly associated with coronary heart disease [29]. HDL-C can prevent CVDs such as arteriosclerosis by removing cholesterol from the blood stream, whereas LDL-C causes accumulation of cholesterol in blood vessels. TC/HDL-C and LDL-C/HDL-C ratios are recognized as two important indicators of CVDs risk with greater predictive value than isolated parameters used independently [30]. The higher the ratios are, the greater the CVDs risk is. Therefore, reduction of serum total cholesterol (TC) and LDL-C and increase of HDL-C may be an important treatment option for CVDs. Probiotics are defined as “living microbial supplements that beneficially affect the host animals by improving its intestinal microbial balances” [31]. In this study, we used two NS *lactobacillus* strains isolated from natural fermented dairy products collected from grassland in Inner Mongolia of China, *Lactobacillus plantarum* NS5 and *Lactobacillus delbrueckii* subsp. *bulgaricus* NS12. We found that NS5 strain could significantly reduce the serum TC and LDL-C levels (reduced by 18.11% and 33.33%, respectively) compared to rats fed with high-cholesterol diet by the end of 6 weeks. Similar results were previously reported in other *Lactobacillus plantarum* strains [12,13]. NS12 strain also reduced the levels of TC and LDL-C (reduced by 12.83% and 22.44%, respectively), but there were no statistical significances compared with HC group. However, there was also no significant difference with NS5 strains. They all didn’t obviously influence HDL-C levels. Meanwhile, both NS5 and NS12 strains significantly reduced the TC/HDL-C (23.25% *vs*. 15.41%) and LDL-C/HDL-C ratios (37.14% *vs*. 23.81%) and NS5 strain seemed more effective than NS12 strain. Liver cholesterol levels were also examined. NS5 and NS12 strains all obviously decreased the cholesterol levels in liver compared with HC group, but contrary to the results of serum TC, NS12 was more effective than NS5 in liver cholesterol lowering activity (34.58% *vs*. 13.47%). These results maybe suggested that NS5 strain was more effective in reducing cholesterol absorption and increasing serum cholesterol removal and NS12 strain was more effective in reducing liver cholesterol synthesis and accumulation.

ApoA-I is the principal apolipoprotein in HDL and ApoB represents most of the protein content in LDL. The apoB/apoA-I ratio is also a strong risk factor for predicting CVDs [32]. Studies even suggested that the ApoB/ApoA-I ratio was better than the TC/HDL-C and LDL-C/HDL-C ratios to estimate the balance between the serum proathergenic and antiatherogenic lipoproteins and to predict CVDs risk [33]. NS5 and NS12 strains displayed significant effects on decreasing ApoB/ApoA-I ratio.

There is an increasing recognition about the potential role for serum free fatty acids (FFA) in CVDs [34]. In this study, we found elevated serum FFA levels in rats fed high-cholesterol diets, and NS5 and NS12 strains significantly decreased the FFA levels. Research found that an increase in serum FFA induced markers of endothelial activation, vascular inflammation and thrombosis and suggested that increases in serum FFA may initiate early vascular abnormalities then promote CVDs [35]. These results suggested that the NS5 and NS12 strains could reduce the risk of incidence of CVDs.

Generally considered, high-cholesterol diets could increase the body weight. In this study, high-cholesterol diets induced the increase of body weight, but did not statistical significant (p = 0.070). NS5 and NS12 slightly decreased the body weight compared with HC group, but also without significant difference. The same results were observed in WAT weights. This may be associated with the dose and method of strains used and the length of treatment. But we still believe NS5 and NS12 strains have a potentiality to reduce the body weight. Meanwhile, we found that high-cholesterol diets obviously increased liver weight and it was significantly reduced by NS5 strains. Liver TG levels were also analyzed, and NS5 and NS12 strains significantly reduced the storage of TG in rats liver. This was also proved by the observation of oil red O staining of liver tissues. Histopathology of liver also suggested severe injuries in liver tissues of high-cholesterol diets fed rats, and NS5 and NS12 strains partially ameliorated the injuries.

Intestinal microbiota modulates host energy and lipid metabolism [6]. Studies also suggested that high-fat feeding induces changes of intestinal microbiota which was associated with an increased intestinal permeability and consequently triggered inflammation and metabolic disorders [36]. Ley et al. reported that the relative abundance of the *Bacteroides* in obese mice was lower, whereas the *Firmicutes* was higher [37]. And the obesity-associated intestinal microbiota has an increased capacity to harvest energy from the diet [38]. The high-cholesterol diet did not alter the proportion of *Firmicutes*, but the proportion of *Bacteroides* was the lowest in rats fed a high-cholesterol diet. NS5 and NS12 strains increased the proportion of *Bacteroides*. The increased body weight in HC group was probably because of the decreased proportion of *Bacteroides* which increased the energy harvesting from the diet. NS5 and NS12 strains increased the proportion of *Bacteroides* and resulted in the reduction of body weight to some extent. We also found *Clostridium* was significantly increased in high-cholesterol diets fed rats, and NS5 and NS12 strains restored the changes. These results suggested that high-cholesterol diets induced the alteration in intestinal microbiota compositions and consequently led to metabolic disorders, and finally resulted in increased serum cholesterol. This also suggested that serum cholesterol-lowering effects of NS5 and NS12 strains may be attributed to the restore of intestinal microbiota compositions by the two NS *lactobacillus* strains. Intestinal microbiota also acts as an environmental factor regulating fat storage [39]. In our study, the WAT weight was the highest in rats fed a high-cholesterol diet, and NS5 and NS12 strains reduced the weight of WAT. This may be also because of the high-cholesterol diets induced the changes in intestinal microbiota compositions. On the other hand, NS5 and NS12 strains also reduced the adipocyte size in rats fed a high-cholesterol diet. Therefore, NS5 and NS12 strains may exert a beneficial effect on the high-cholesterol diets induced increase in WAT weight partly by reducing the cell size of adipocyte.

The serum cholesterol level is regulated by three distinct pathways: absorption, synthesis and excretion. LDLR is a receptor on the hepatocyte surface that mediates the endocytosis of LDL-C and the mRNA expression level of LDLR was negatively correlated with cellular cholesterol content [40]. High cholesterol diets significantly decreased the mRNA expression level of LDLR, therefore resulted in increase of serum LDL-C level. NS5 strain did not alter LDLR expression level and NS12 strain slightly increased mRNA expression level of LDLR. But NS5 strain seemed more effective in reducing LDL-C level than NS12 strain, maybe other mechanisms involved in this process. HMG-CoA reductase is the rate-limiting enzyme in hepatic cholesterol *de novo* synthesis, catalyzing the conversion of HMG-CoA to mevalonic acid [41]. The liver HMG-CoA reductase expression is correlated with the rate of cholesterol synthesis and the abundance of cholesterol sources from diet would inhibit HMG-CoA reductase activity [42]. The mRNA expression level of HMG-CoA reductase was significantly reduced in high cholesterol diets fed rats comparing to rats fed normal diets, and the inhibitory effects of NS5 and NS12 strains seemed more obvious, especially NS5 strain. Therefore, the reduction of serum cholesterol levels by NS *lactobacillus* strains may be partly through inhibiting HMG-CoA reductase expression levels. Spady *et al*. reported that over expression of CYP7A1 gene effectively reduced the serum TC and LDL-C level in hamsters fed a high-fat diet [43]. In the present study, the liver CYP7A1 mRNA level was upregulated higher by NS5 and NS12 strains supplementation in the drinking water than rats fed high-cholesterol diets alone. This may be the mechanism by which NS5 and NS12 strains could reduce serum TC and LDL-C. ACAT is the enzyme which catalyzes the 3-OH group of cholesterol fatty-acylated to form cholesterol esters that can be stored in cytoplasmic lipid droplets [44]. NS5 and NS12 strains significantly inhibited ACAT mRNA expression and reduced cholesterol stored in the body in the form of cholesterol esters.

## Conclusions

The two NS *lactobacillus* strains, *Lactobacillus plantarum* NS5 and *Lactobacillus delbrueckii* subsp. *bulgaricus* NS12, exerted significant serum cholesterol lowering effects on male SD rats fed a high-cholesterol diet. We explored the possible mechanisms from the aspects of intestinal microbiota compositions and mRNA expression levels of liver enzymes related to cholesterol metabolism, but further studies are needed to elucidate the precise mechanism. The NS *lactobacillus* strains may become more effective and safer alternative therapies to lower serum cholesterol.

## Abbreviations

CVD: Cardiovascular disease; LAB: Lactic acid bacteria; WAT: White adipose tissues; HC: High cholesterol; TC: Total cholesterol; TG: Triglyceride; HDL-C: High density lipoprotein cholesterol; LDL-C: Low density lipoprotein cholesterol; ApoA-I: Apolipoprotein A-I; ApoB: Apolipoprotein B; FFA: Free fatty acid; GAPDH: Glyceraldehyde-3-phosphate dehydrogenase; HMG-CoA R: 3-hydroxy-3-methylglutaryl-CoA reductase; LDLR: Low density lipoprotein receptor; CYP7A1: Cholesterol 7α-hydroxylase; ACAT: Acyl-CoA:cholesterol acyltransferase; LCAT: Lecithin cholesterol acyltransferase.

## Competing interests

All authors declare that they have no competing interests.

## Authors’ contributions

HX, WT and LW performed experiments, data collection, statistical analysis and wrote the manuscript. JF and WL designed and conducted the study and wrote the manuscript. HX and WT contributed to the work equally and should be regarded as co-first authors. JF and WL should be regarded as co-corresponding authors. All authors read and approved the final manuscript.
